# Anti-seizure effects of WS-3, a TRPM8 agonist, on focal onset seizure mouse model via reduction of extracellular glutamate levels

**DOI:** 10.1038/s41386-025-02143-x

**Published:** 2025-06-26

**Authors:** Hiroshi Moriyama, Sadahiro Nomura, Hirochika Imoto, Yuichi Maruta, Naomasa Mori, Natsumi Fujii, Kohei Haji, Michiyasu Suzuki, Hideyuki Ishihara

**Affiliations:** 1 Department of Neurosurgery, Graduate School of Medicine, Yamaguchi University, Ube, Yamaguchi, Japan; 2 Epilepsy Center, Yamaguchi University Hospital, Ube, Yamaguchi, Japan

**Keywords:** Pharmacology, Ion channels in the nervous system

## Abstract

The development of novel anti-seizure drugs targeting novel mechanisms is crucial, especially for patients with intractable epilepsy. Previous studies using focal onset seizure rodent models have demonstrated that Icilin and WS-3, agonists of the transient receptor potential melastatin 8 (TRPM8) channel, suppress drug-induce epileptiform discharges (EDs) and seizures (ESs). In contrast, TRPM8 deficiency exacerbates EDs and ESs. This study investigated the mechanism underlying the anti-seizure effects of the TRPM8 agonist, WS-3, using a focal onset seizure mouse model. Mice were injected with WS-3 either before or after administering the seizure inducer, penicillin G potassium. EDs, ESs, and glutamate levels were subsequently evaluated. In wild-type (WT) mice, WS-3 injected after the seizure inducer reduced glutamate levels and ED power by 44% and 60%, respectively, with a positive correlation between WS-3 efficacy and these parameters. WS-3 injection before seizure induction suppressed the increase in glutamate levels and the development of ED and ES, with positive correlations observed among the three parameters. Conversely, TRPM8-knockout mice showed no anti-seizure effects from WS-3. TRPM8 deficiency led to a further increase in the glutamate levels, ED power, and ES severity after the seizure inducer injection. Additionally, TRPM8-deficient mice experienced EDs with fewer glutamate exposures and shortened latency to ED development following seizure induction. These findings suggest that TRPM8 agonists suppress the development of EDs and ESs by reduction of extracellular glutamate levels, indicating that TRPM8 channels may represent a promising treatment option for epilepsy.

## Introduction

Epilepsy is a prevalent neurological disorder that affects the central nervous system, with approximately 30% of cases classified as intractable. Intractable epilepsy is characterized by seizures that cannot be inadequately controlled even with the use of multiple anti-seizure drugs or changing therapeutic agents [1–3]. The difficulty in managing intractable epilepsy highlights the need for developing new anti-seizure drugs based on novel mechanisms of action.

Transient receptor potential melastatin 8 (TRPM8) agonists, such as Icilin and WS-3, have shown promise in suppressing drug-induced epileptiform discharges (EDs) in focal-cerebral cortex onset seizure model rodents, suggesting that TRPM8 channels could serve as a novel therapeutic target for epilepsy [4, 5]. TRPM8 channels are expressed in human glial cells and trigeminal ganglion neurons [6, 7], and to a lesser extent, in the rodent brain, particularly in regions such as the cerebral cortex and hippocampus [8]. These channels are activated by TRPM8-selective agonists [9] or by cold temperatures ranging from 10–26 °C in peripheral cold-sensing neurons [10]. Cooling the epileptic focus to approximately 15 °C has been shown to suppress EDs in patients with focal refractory epilepsy [11, 12], possibly through TRPM8 activation, indicating that TRPM8 activation could trigger anti-seizure effects. However, the precise mechanisms underlying these TRPM8-mediated effects are not fully understood.

One of the mechanisms of epilepsy involves the elevation of extracellular glutamate levels, which contributes to neuronal hyperexcitability. Studies on patients with focal-intractable epilepsy have shown that focal brain cooling to 15 °C [11, 12], a temperature that activates TRPM8 channels, attenuates extracellular glutamate levels. TRPM8-expressing cells in the lateral septum (LS) and preoptic area (POA) are primarily inhibitory γ-aminobutyric acid (GABA)-ergic neurons [13], and TRPM8 agonists have been found to suppress excitatory post-synaptic glutamatergic neurons [14]. These findings suggest that TRPM8 agonists could regulate extracellular glutamate levels by modulating GABAergic cells. Despite these insights, it remains difficult to determine the exact relationship between TRPM8 activation and extracellular glutamate concentrations because of the limitations of conventional techniques. Epileptiform spikes are extremely brief (< 100 ms), while glutamate measurements using microdialysis operate with a time resolution on the order of minutes.

To address this challenge, the present study used a biosensor capable of real-time glutamate measurement, allowing for a more accurate assessment of the relationship between TRPM8 activation and extracellular glutamate levels [15]. Penicillin G (PG)-induced experimental seizure, a well-established model mediated by GABA_A_-receptor antagonists [4, 5, 16–18], was employed. In a previous study, a microdialysis probe inserted into a small area of the seizure focus measured the glutamate concentration [12]. However, to obtain a more representative measurement of the entire focal area, a more advanced technique is needed. The PG-induced focal onset seizure model is particularly useful for evaluating extracellular glutamate regulation, as preventive administration of WS3 at the seizure focus has been shown to suppress both EDs and seizures [5].

Since Icilin is a non-selective TRPM8 and TRP Ankyrin 1 agonist [14], we chose WS-3, a more selective TRPM8 agonist, for use in this study to better isolate the effects of TRPM8 activation. We conducted two studies to clarify whether the suppressive effects of WS-3 on focal EDs and seizures are mediated via the regulation of extracellular glutamate levels. In the first study, we evaluated the correlation between WS-3 efficacy, ED power, and extracellular glutamate levels by administering WS-3 directly into the seizure focus 60 min after the seizure inducer injection. In the second study, we assessed the effects of WS-3 on EDs, epileptiform seizures (ESs), and extracellular glutamate levels by injecting WS-3 30 min before the seizure inducer. Finally, we compared the effects of the seizure inducer and WS-3 between wild-type (WT) and TRPM8-knock out (KO) mice to examine whether TRPM8 deficiency exacerbates ED power, ES severity, and extracellular glutamate levels and whether the anti-seizure effects of WS-3 are absent in TRPM8-KO mice.

## Material and methods

### Animals

All experiments were conducted using a modified version of a previously reported method [5]. Male C57BL/6 N and TRPM8-KO mice (kindly provided by Prof. Makoto Tominaga, Thermal Biology Group, Exploratory Research Center on Life and Living Systems, Okazaki, Japan) aged 9–11 weeks, weighing 24–30 g (Kyudo, Saga, Japan) [19], were housed in groups of five per cage and kept under standard laboratory conditions. These conditions included a temperature- and humidity-controlled room (25 ± 2 °C and 55 ± 5%, respectively) with a 12-hour light/dark cycle (lights on at 8:00 a.m.). The animals had *ad libitum* access to food and water. Animal care and experimental procedures were approved by the Experimental Animal Care and Use Committee of the Yamaguchi University School of Medicine, Ube, Japan (Approval number: J18030 and J23053). All experiments were performed in accordance with the guidelines of the Japan Association for Laboratory Animal Facilities of the National University Corporation and reported in accordance with the ARRIVE guidelines.

### ECoG recordings

ECoG recordings were performed following a previously reported method [5]. The animals were anesthetized with urethane (1.75 g/kg i.p.) or sevoflurane (Pfizer Japan, Tokyo, Japan) (3% for induction and 1.5% for maintenance) and immobilized using a stereotaxic apparatus. Body and brain temperatures were maintained at 37.5 ± 0.5 °C using a heating pad. Three burr holes (1.0 mm in diameter) were made: two for the ECoG recording above the bilateral sensorimotor cortices and one above the cerebellum for the reference electrode. The coordinates used were 1.0 mm posterior and ±2.0 mm lateral from the bregma, and 2.0 mm posterior from the lambda. A thin thermocouple (IT-24; Physitemp, Tokyo, Japan), two ECoG electrodes, and a reference electrode were placed between the skull and dura. The ground electrode was positioned on the tail, and the heart rate was monitored using electrocardiography to estimate the depth of anesthesia.

### Drugs

Penicillin G potassium (PG; Meiji, Tokyo, Japan) induced experimental seizure is a model of seizure mediated by a non-competitive GABAA-receptor inhibitors [4, 5, 17, 20–22]. PG is an effective seizure inducer for the development of anti-seizure medications because it frequently and continuously induces EDs at least 90 min under anesthesia [5]. We chose a focal onset seizure model to reflect focal to bilateral tonic-clonic seizures, which are the most common seizures in epilepsy. PG was dissolved in saline [23, 24]. *N*-Ethyl-5-methyl-2-(1-methylethyl)cyclohexanecarboxamide (WS-3; Funakoshi, Tokyo, Japan), a TRPM8 agonist, at a concentration of 1 µM mitigated PG-induced ED development and ES severity [5]. Modifying the previous report, 1 µM WS-3 was prepared using 1% dimethyl sulfoxide (DMSO; Merck KGaA, Darmstadt, Germany) in phosphate buffered saline [5].

### Generation of the drug-induced focal onset seizure model and drug treatments

To create the model with focal onset seizure originating in the left somatosensory cortex, a previously reported method was followed with some modifications [5]. PG and WS-3 were injected into the same cortical location but *via* separate routes, as described previously [5]. A 10 μL Hamilton syringe with a 26-gauge removable needle (1701RN-7758-02; Hamilton, Reno, NV, USA) was used. The inner cavity of the removable needle was filled with 1 μL of PG (200 IU/μL, dissolved in 0.9% saline), while the 10 μL syringe body was filled with 1 μL of PG and 8.6 μL of WS-3, which were separated by 0.4 μL of air. The injection cannula and Hamilton syringe were then connected via a Teflon tube (JT-10; 50 cm-long, 4 μL volume; Eicom, Kyoto, Japan). After the PG in the Hamilton syringe reached the tip of the injection cannula (0.4 mm diameter × 40 mm length, and 3 µL volume; EIM-40, Eicom) through the Teflon tube, the injection cannula was inserted 0.75 mm from the brain surface on the left sensorimotor cortex. PG and WS-3 were injected intracortically for 10 min using a microinjection pump at a rate of 0.1 µL/min (ESP-64, Eicom).

### Drug treatments

In the first half of the study, WS-3 was administered 60 min after PG injection to evaluate the effects of the TRPM8 agonist on EDs and extracellular glutamate levels. In the second half, mice were treated with DMSO or WS-3 30 min before PG injection to assess the preventive effects of the TRPM8 agonist on ED development, ESs, and the elevation of extracellular glutamate levels induced by the epileptic inducer.

### ECoG analysis

ECoGs were recorded by modifying a previously reported method [5]. EDs typically developed 10–20 min after PG injection, reached a steady state 1 h after injection [5], and remained stable for at least 5 h (data not shown). ECoGs were continuously recorded for 100 min (10 min for ECoG stabilization, 60 min for stabilization of PG-induced EDs, and 30 min for drug efficacy evaluation in the post-injection stage, and 10 min for ECoG stabilization, 30 min for ECoG stabilization after 1% DMSO or WS-3 injection, and 60 min for stabilization of PG-induced Eds in the pre-injection stage). The ECoGs were amplified using a bioamplifier (EX-1; Dagan Corporation, Minneapolis, MN, USA) with an analog-to-digital converter, at a sampling rate of 2 kHz (PowerLab 8/30; AD Instruments, Castle Hill, Australia). The conditions for recording ECoGs were as follows: low-frequency filter, 0.1 Hz; high-frequency filter, 10 kHz; and notch filter, off.

### Drug efficacies

In the post-injection study, WS-3 eliminated EDs within minutes [5], and the efficacy period was considered the 5–10 min interval after TRPM8 agonist injection. In the pre-injection study, efficacy was evaluated during the 55–60 min interval after seizure inducer injection. To assess the effects of TRPM8 channel activation on PG-induced EDs, the left ECoGs were fast Fourier-transformed. The power of the low beta-band (14–24 Hz) was calculated using LabChart Pro version 8.1.21 (AD Instruments). Changes in the low beta-band power in the ECoG were used as indicators of the degree of focal neocortical seizures and the suppression of EDs by focal brain cooling and WS-3 administration [5, 17, 25, 26]. Beta-band power also correlates with magnetic resonance fingerprinting T_1_ images in patients with pharmaco-resistant epilepsy and abnormalities of the brain network organization in focal epilepsy [27, 28].

### Seizure score

Mouse behaviors were observed for 1 h following the 100 min ECoG recording. PG-induced seizure intensity was scored as follows, based on previous studies [29]: stage 0, no response; stage 1, ear and facial twitching; stage 2, convulsive twitching axially through the body; stage 3, myoclonic jerks and rearing; stage 4, turning over onto the side, running, and jumping; stage 5, generalized tonic-clonic seizures; and stage 6, death.

### Extracellular glutamate recordings

L-glutamate biosensors allow for real-time recording by harnessing the innate efficiency, activity, and specificity of the biological recognition element (L-glutamate oxidase), to provide high sensitivity [15, 30]. Briefly, the extracellular brain level of glutamate was monitored using a platinum enzyme-linked electrode (7011; Pinnacle Technology, Lawrence, KS, USA). The sensor was prepared from Pt-Ir wires with diameters of 180 µm, wrapped concentrically with an AgCl reference electrode. L-Glutamate oxidase (EC 1.4.3.11) in the sensing cavity oxidizes the analyte of interest, resulting in an equivalent release of hydrogen peroxide [15]. Modifying a previously reported method, hydrogen peroxide was subsequently detected and recorded as an amperometric oxidation voltage by the electrode, using a 16-bit PowerLab 8/30 system and LabChart version 8.21 software.

**Fig. 1:**
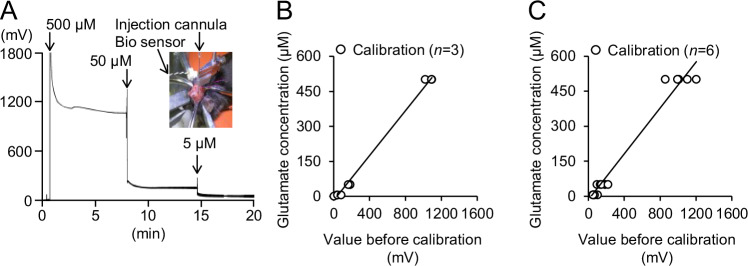
Calibration of extracellular glutamate levels from voltage levels to concentrations using standard solutions. **A** Voltage levels of extracellular glutamate (standard solutions: 0, 5, 50, and 500 μM) were corrected to concentrations using pre-calibration titration lines. The correlation diagram shows results from experiments where the TRPM8 agonist was injected (**B**) after and (**C**) before the seizure inducer injection. In each case, the pre-calibration titration lines were generated from three and six recordings, respectively. The linear functions were *y* = 0.4804*x* – 17.173 and *y* = 0.0492*x* – 15.023 (*R*^*2*^ = 0.9939 and 0.9771, respectively).

To accurately record extracellular glutamate levels, the seizure inducer, PG, was injected into the somatosensory cortex of mice, followed by the injection of WS-3 at the seizure focus. In the preliminary verification, standard solutions (5, 50, 100, 250, and 500 μM) allowed for the accurate conversion of voltage levels into glutamate concentrations (*y* [µM] = 0.624*x* [mV] –0.997 [mV], R^2^ = 0.9951, *p* < 0.001, 95% confidence interval, CI: 0.9768, 0.9997; Fig. S1). After the craniotomy, the sensor was inserted 0.75 mm below the dural surface. Before the extracellular glutamate levels were recorded, the data was calibrated using standard solutions (0, 5, 50, and 500 μM). To confirm the validity of the unit conversions, correlation coefficients were calculated by the pre-calibration titration lines. The sensor responded to glutamate linearly at 30 °C, in 0.1 M phosphate-buffered saline (pH 7.4) (Fig. 1).

A 16-bit PowerLab 8/30 was used as the analog-to-digital converter. All signals were recorded and converted from mV to μM using LabChart Pro version 8.1.25 (AD Instruments, Oxford, UK). The L-glutamate levels were averaged every second by the biosensors [15].

### Statistical analysis

All data are presented as mean ± standard error of the mean. Statistically significant differences were evaluated using paired *t*-tests, Student’s *t*-tests, Welch’s *t*-tests, Dunnett’s test, or Tukey’s tests, with *p* < 0.05 indicating statistical significance. JMP Pro 16.1.0 for Windows (SAS Institute, USA) was used for statistical analysis.

For the TRPM8 agonist injection after the seizure inducer injection, data between two groups (PG + WS-3 in W.T. and TRPM8KO mice) were analyzed using Student’s or Welch’s *t*-tests. TRPM8 agonist efficacy within the same group was analyzed using paired *t*-tests. The correlation extent of two parameters was indicated by Pearson’s correlation coefficients. In the pre-injection study, data among four groups (DMSO + PG / W.T., WS-3 + PG / W.T., DMSO + PG / TRPM8KO, and WS-3 + PG / TRPM8KO), excluding seizure score data, were analyzed using Tukey’s test. Seizure scores were valued by Steel-Dwass test after Kruskal-Wallis test because seizure scores are a discrete variable. The correlation extent of two parameters, excluding seizure scores, was indicated by Pearson’s correlation coefficients. The correlation coefficients including seizure scores were valued using Spearman’s rank order correlation coefficients because seizure scores are a discrete variable.

## Results

### Calibration of extracellular glutamate levels

A biosensor was used to record the extracellular glutamate levels in real-time [15]. To prepare a calibration curve, glutamate solutions were prepared at varying concentrations and recorded by the biosensor (Fig. 1A). By modifying a previously published method, the voltage level of the extracellular glutamate was translated into concentration [15]. The pre-calibration titration lines allowed for the accurate conversion of voltage levels into glutamate concentrations [*y* (µM) = 0.4804*x* (mV) –17.173 (mV) and *y* (µM) = 0.0492*x* (mV) – 15.023 (mV), *R*^2^ = 0.9939, *n* = 3 and R^2^ = 0.9771, *n* = 6 respectively; Fig. 1B, C].

### A TRPM8 agonist, WS-3, decreases glutamate levels and ED power

A previous study with focal onset seizure model mice demonstrated that WS-3 suppressed drug-induced EDs, while TRPM8 deficiency exacerbated them [5]. Since TRPM8 channels are expressed in inhibitory GABAergic neurons [13], TRPM8 agonists are expected to regulate neuronal excitability. In this study, we investigated the extent to which TRPM8 agonist efficacy correlates with PG-induced changes in extracellular glutamate levels and EDs.

**Fig. 2:**
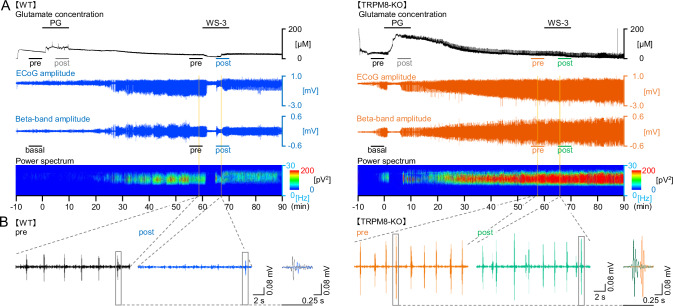
Representative effects of WS-3, the TRPM8 agonist, on extracellular glutamate levels and beta-band power in WT and TRPM8-KO mice after the seizure inducer injection. **A** Representative changes in glutamate levels, ECoG amplitudes, Beta-band amplitudes, and beta-band power in wild-type (WT) and TRPM8 knockout (TRPM8-KO) mice. **B** Traces are highlighted by yellow columns. ECoGs at 20 s and 1 s show representative beta-band amplitudes before and after TRPM8 agonist injection in each group. ECoG, electrocorticogram; PG, penicillin G potassium (seizure inducer); TRPM8, transient receptor potential melastatin 8; TRPM8-KO, *TRPM8* homozygous knockout; WT, wild type.

We only compared the effects of WS-3 on PG-induced changes in glutamate levels and EDs between WT and TRPM8-KO mice because we found that DMSO did not affect these parameters (Fig. S2). The seizure inducer increased glutamate levels, ECoG amplitude, beta-band amplitude, and ED power (Fig. 2A). WS-3 decreased glutamate levels, ED power, and beta-band amplitude in WT mice, while no anti-seizure effects were observed in TRPM8-KO mice (Fig. 2A). Magnified traces (yellow columns) highlight the effects of WS-3 on ED power (Fig. 2B).

**Fig. 3:**
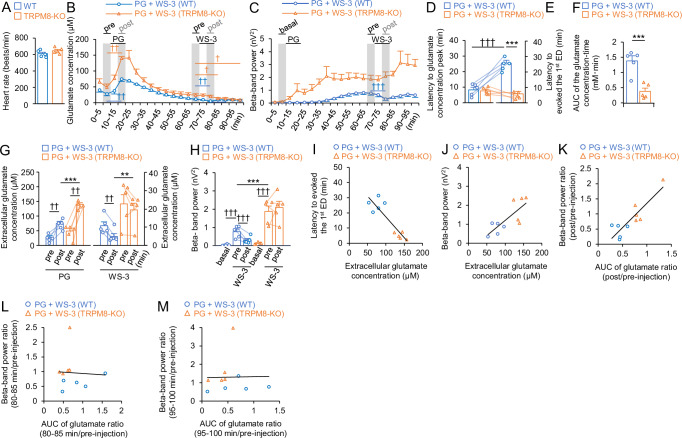
WS-3, the TRPM8 agonist, decreased extracellular glutamate levels and beta-band power in WT mice after the seizure inducer injection; however, WS-3 did not decrease in TRPM8-KO mice. **A** Average heart rate in each group. **B** Glutamate level transitions averaged every 5 min; *n* = 5 for each group. Gray columns indicated each evaluation time zone (pre-PG: 5–10 min, post-PG: 15–20 min, per-WS-3: 65–70 min, and post-WS-3: 75–80 min). **C** Beta-band power transitions averaged every 5 min; *n* = 5 for each group. Gray columns indicated each evaluation time zone (basal: 5–10 min, per-WS-3: 65–70 min, and post-WS-3: 75–80 min). **D** Time taken to reach peak glutamate levels. **E** Latency to the first ED. **F** Area under the glutamate concentration-time curve, until the first ED. **G** Glutamate levels for each gray column in *B*, before and after the seizure inducer and TRPM8 agonist injections (pre-PG: 5–10 min, post-PG: 15–20 min, per-WS-3: 65–70 min, and post-WS-3: 75–80 min). **H** Beta-band power for each gray column in *C*: basal activity before and after WS-3, a TRPM8 agonist, injection (basal: 5–10 min, per-WS-3: 65–70 min, and post-WS-3: 75–80 min). Correlation between **I** peak glutamate levels and latency to the 1st ED (*R* ^*2*^ = 0.800), **J** peak glutamate levels and beta-band power, at 55–60 min after the seizure inducer injection (*R*^*2*^ = 0.600), and (**K**) efficacy of the TRPM8 agonist on glutamate levels and beta-band power (*R*^*2*^ = 0.759). In the period, the glutamate level and beta-band power were decreased by WS-3 administrated in WT mice (**B**-**C**). Correlation between efficacy of the TRPM8 agonist on glutamate levels and beta-band power during (**L**) 0-5 and (*M*) 15–20 min after the end of WS-3 administration (*R*^*2*^ = 0.00289 and *R*^*2*^ = 0.000372, respectively). In each period, only the glutamate level was decreased by WS-3 administrated in TRPM8-KO mice (**B**, **C**). Opened blue circles and orange triangles indicate (**A**, **D**–**M**) raw data or (**B**, **C**) mean in each group. Data in (**A**–**H**) are presented as mean ± SEM (*n* = 5 mice per group). *p < 0.05 and ***p < 0.001, Student’s *t*-test. ^†^*p* < 0.05, ^††^*p* < 0.01 and ^†††^*p* < 0.001, paired *t*-test. ECoG, electrocorticogram; EDs, epileptiform discharges; PG, penicillin G potassium (seizure inducer); TRPM8, transient receptor potential melastatin 8; TRPM8-KO, *TRPM8* homozygous knockout; WT, wild type.

Heart rate data indicated no significant difference in the depth of anesthesia between the groups (*n* = 5 per group; 609.72 ± 18.21 vs. 646.20 ± 16.00, for the PG + WS-3 in WT and TRPM8-KO mice groups, respectively; *p* = 0.1714; Fig. 3A). To determine the effects of the TRPM8 agonist on glutamate levels and ED power, extracellular glutamate levels and the power of the beta-band in the EDs were evaluated for the WT and TRPM8-KO mice (Fig. 3B–K). The transitions in glutamate levels and ED power every 5 min differed between the WT and TRPM8-KO mice (Fig. 3B, C). The glutamate levels before PG injection were not different between WT and TRPM8-KO mice (0-5 min, *p* = 0.0653 and 5–10 min, *p* = 0.1045, respectively, Student’ *t*-test; Fig. 3B). In WT mice, the latency to reach maximum glutamate levels was 8.38 ± 1.71 min (Fig. 3D), which subsequently induced the development of EDs (25.72 ± 1.84 min, *p* < 0.0001, paired *t*-test; Fig. 3E). The area under the curve until the first ED was 1.36 ± 0.17 mM/min (Fig. 3F). Glutamate levels and ED power increased 10 and 60 minutes after PG injection, respectively, in WT mice, while WS-3 significantly decreased both parameters by 44% and 60%, respectively (Glutamate levels: pre-PG: 27.56 ± 5.51 µM, post-PG: 74.65 ± 7.43 µM, *p* = 0.0049, and pre-injection: 10.97 ± 2.16 µM, post-injection: 5.03 ± 1.60 µM, *p* = 0.0028, paired *t*-test; beta-band power: basal: 0.0396 ± 0.0151 nV^2^, pre-injection: 0.762 ± 0.145 nV^2^, post-injection: 0.304 ± 0.083 nV^2^, *p* = 0.0095 and *p* = 0.0313, respectively, paired *t*-test; Fig. 3G, H). These effects were absent in TRPM8-KO mice (Glutamate levels: pre-injection: 23.15 ± 5.16 µM, post-injection: 19.85 ± 3.69 µM, *p* = 0.2582; beta-band power: pre-injection: 1.87 ± 0.30 nV², post-injection: 2.09 ± 0.34 nV², *p* = 0.5248, paired *t*-test; Fig. 3G, H). Data from both groups showed that higher peak glutamate levels were associated with shorter latencies to ED development (*r* = −0.895, *R*^2^ = 0.800, *p* < 0.001, 95% confidence interval, CI: −0.975, −0.606, Pearson’s correlation coefficients; Fig. 3I). Furthermore, higher peak glutamate levels were correlated with increased ED power, and the effects of WS-3 on glutamate levels and ED power were positively correlated (R^2^ = 0.6001 and 0.7587, respectively; Fig. 3J, K).

### TRPM8 deficiency causes glutamate levels and ED power to increase further

Since TRPM8 agonists activate GABAergic neurons [13], and TRPM8 deficiency has been shown to exacerbate EDs [5], we hypothesized that TRPM8 deficiency would further elevate glutamate levels following seizure induction. To confirm this, we compared extracellular glutamate levels and ED power between WT and TRPM8-KO mice. TRPM8 deficiency did not significantly affect the time required to reach maximum glutamate levels (7.72 ± 1.02 min, *p* = 0.752; Fig. 3D). However, TRPM8-deficient mice developed EDs with fewer glutamate exposures, leading to shorter latencies to ED development (0.38 ± 0.11 mM/min, *p* = 0.0011; 5.00 ± 1.00 min, *p* < 0.0010, respectively; Fig. 3E-F). Additionally, TRPM8 deficiency significantly increased glutamate levels 5–10 min post-injection (139.84 ± 5.57 µM, *p* < 0.0001) and ED power 55–60 min post-injection (1.87 ± 0.30 nV^2^, *p* = 0.0163) compared to WT mice (Fig. 3G, H). In TRPM8-KO mice, the reductions of WS-3 on glutamate levels were observed at 5 and 20 min after the reduction in WT mice (80–85 min: 13.16 ± 3.04 µM, *p* = 0.0261, and 95–100 min: 8.60 ± 2.34 µM, *p* = 0.0308, respectively, paired *t*-test, vs 65–70 min: 23.15 ± 5.16 µM; Fig. 3B). In contrast, beta-band power was not changed by WS-3 during 0-5 and 15-20 min after the end of WS-3 administration in TRPM8-KO mice (Beta-band power: pre-injection: 1.87 ± 0.30 nV², 80–85 min: 2.21 ± 0.269 nV², 95–100 min: 2.93 ± 0.468 nV², *p* = 0.3389 and *p* = 0.1269 vs pre-injection, respectively, paired *t*-test; Fig. 3C). During the two periods, the efficacy of WS-3 on glutamate levels and ED power was not positively correlated (*r* = −0.0537, *R*^2^ = 0.00289, *p* = 0.883, 95% confidence interval, CI: −0.661, −0.596; and *R*^2^ = 0.000372, *p* = 0.958, 95% confidence interval, CI: −0.618, −0.641, respectively, Pearson’s correlation coefficients; Fig. 3L-M).

### TRPM8 agonist pre-injection reduces an increase in glutamate levels and the development of EDs and ESs

TRPM8 agonists have been shown to reduce ED power and seizure severity [5]. The efficacies of the TRPM8 agonist on EDs and glutamate levels were positively correlated (Fig. 3K). This suggests that TRPM8 agonists reduce glutamate level increases following seizure induction and subsequently suppress the development of EDs and seizure severity. In the second half of the study, we investigated the preventive effects of WS-3 on glutamate levels, ED development, and seizure severity. We compared the WT/DMSO + PG, WT/WS-3 + PG, TRPM8-KO/DMSO + PG, and TRPM8-KO/WS-3 + PG groups. To evaluate the preventive effects of WS-3, the TRPM8 agonist was injected 30 min before PG administration in the same somatosensory cortex location. All 18 mice were recorded for electroencephalograms, glutamate levels, and seizures (WT/DMSO + PG, *n* = 5; WT/WS-3 + PG, *n* = 5; TRPM8-KO/DMSO + PG, *n* = 4; TRPM8-KO/WS-3 + PG, *n* = 4).

**Fig. 4:**
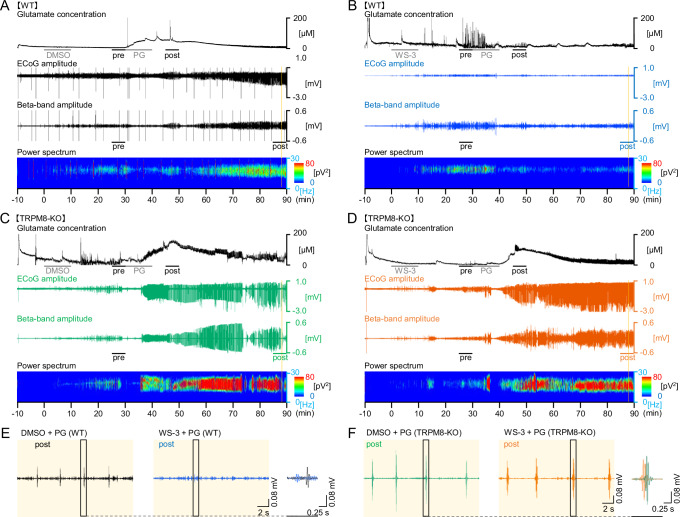
Representative effects of WS-3, the TRPM8 agonist on extracellular glutamate levels, beta-band power, and seizure severity in WT and TRPM8-KO mice before the seizure inducer injection. **A**–**D** Representative changes in glutamate levels, ED amplitudes, and beta-band power in the WT and TRPM8-KO mice. **E**, **F** Magnified traces from (**A***,* **B**) are indicated by the yellow columns. ECoGs at 20 s and 1 s showing beta-band amplitudes during the last 5 min. DMSO, dimethyl sulfoxide; ECoG, electrocorticogram; PG, penicillin G potassium (seizure inducer); TRPM8, transient receptor potential melastatin 8; TRPM8-KO, *TRPM8* homozygous knockout; WT, wild type. PG.

Representative changes in glutamate levels and ED power for each group are shown in Fig. 4A–D. Magnified EDs showed that pre-injection of WS-3 in WT mice reduced ED development following PG injection, while this effect was not observed in TRPM8-KO mice (Fig. 4E, F).

**Fig. 5:**
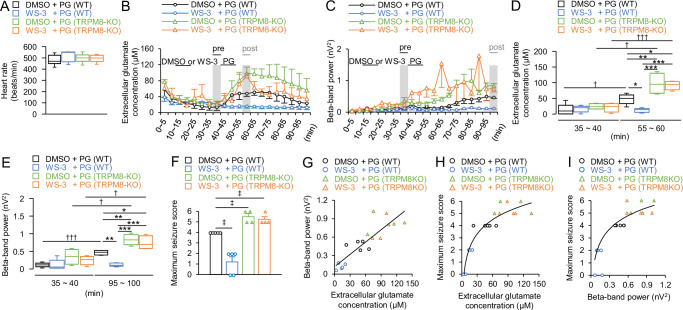
Preventive administration of WS-3, the TRPM8 agonist, reduced the elevation of extracellular glutamate levels, beta-band power, and seizure severity in WT mice after the seizure inducer injection; however, WS-3 did not decrease in TRPM8-KO mice. **A** Average heart rate in each group. Transitions in (**B**) glutamate levels and (**C**) beta-band power were averaged every 5 min. **B***,* **C** Gray columns indicated each evaluation time zone (*B*; pre-PG: 35–40 min, post-PG: 55–60 min and *C*; pre-PG: 35–40 min, post-PG: 95–100 min). **D** Glutamate levels in each gray column in *B*: 5 min before and 15–20 min after the seizure inducer injection. **E** Beta-band power 5 min before the seizure-inducer injection and during the last 5-min period. **F** Maximum seizure score during the 60 min after the end of inhalation anesthesia. Correlation between (**G**) peak glutamate levels and beta-band power (*R*^*2*^ = 0.754), **H** peak glutamate levels and maximum seizure score (*ρ* = 0.905), and (**I**) logarithmic value of beta-band power during the last 5 min of anesthesia and the maximum seizure score (*ρ* = 0.908). Opened black or blue circles and green or orange triangles indicate (**B**, **C**) mean or (**F**–**I**) raw data in each group. Data in (**A**–**F**) are presented as the mean ± SEM, *n* = 5 WT mice and *n* = 4 TRPM8-KO mice. *p < 0.05, **p < 0.01, and ***p < 0.001, Tukey’s test. ^†^*p* < 0.05 and ^†††^*p* < 0.001, paired *t*-test. ^‡^*p* < 0.05, Steel-Dwass test after Kruskal-Wallis test. DMSO, dimethyl sulfoxide; EDs, epileptiform discharges; PG, penicillin G potassium; TRPM8, transient receptor potential melastatin 8; TRPM8-KO, *TRPM8* homozygous knockout; WT, wild-type.

Heart rate data confirmed no significant differences in the depth of anesthesia between the groups (496.82 ± 25.21, 492.88 ± 20.32, 480.50 ± 23.31, and 511.31 ± 24.71, respectively, *p* > 0.7708, Tukey’s test; Fig. 5A). The transitions in glutamate levels and ED power every 5 min differed between the WT and TRPM8-KO mice (Fig. 5B, C). The glutamate levels before DMSO, WS-3, or PG injection did not differ between WT and TRPM8-KO mice (0–5 min, *p* ≥ 0.1848 and 25–30 min, *p* ≥ 0.4379, respectively, Tukey’s test; Fig. 5B). Pre-injection of WS-3 in WT mice resulted in a smaller increase in the glutamate levels and a reduction in EDs and maximum seizure scores (47.80 ± 7.21 µM vs. 11.97 ± 3.03 µM, *p* = 0.0469, Tukey’s test; 0.445 ± 0.078 nV^2^ vs. 0.121 ± 0.049 nV^2^, *p* = 0.0029, Tukey’s test; 4.00 ± 0.00 vs. 1.20 ± 0.490, *p* = 0.0329, Steel-Dwass test after Kruskal-Wallis test, respectively; Fig. 5D, F). In contrast, WS-3 had no significant effect in TRPM8-KO mice (97.21 ± 16.78 µM vs. 91.74 ± 7.01 µM, *p* = 0.9772, Tukey’s test; 0.829 ± 0.077 nV^2^ vs. 0.739 ± 0.097 nV^2^, *p* = 0.7326, Tukey’s test; 5.50 ± 0.58 vs. 5.25 ± 0.50, *p* = 0.9562, Steel-Dwass test after Kruskal-Wallis test, respectively; Fig. 5D, F). In both groups, higher peak glutamate levels were associated with higher ED power and seizure scores (*R*^2^ = 0.754, *p* < 0.001, 95% CI: 0.675, 0.950, Pearson’s correlation coefficients and *ρ* = 0.905, *p* < 0.001, Spearman’s correlation coefficients, respectively; Fig. 5G, H). In addition, a higher ED power correlated with a higher seizure score (*ρ* = 0.908, *p* < 0.001, Spearman’s correlation coefficients; Fig. 5I).

### TRPM8 deficiency increases glutamate levels and exacerbates seizure events

TRPM8 deficiency has been shown to exacerbate EDs and ESs [5] and to further elevate glutamate levels following seizure induction (Fig. 3G). These findings suggest that TRPM8 deficiency increases the severity of epileptic events by more easily elevating extracellular glutamate levels. To confirm this, we compared extracellular glutamate levels, ED power, and seizure severity between WT and TRPM8-KO mice. TRPM8 deficiency significantly increased glutamate levels, ED power, and seizure severity after PG injection (DMSO + PG / WT vs DMSO + PG / TRPM8-KO group, *p* = 0.0089, Tukey’s test; *p* = 0.0030, Tukey’s test; *p* = 0.0492, Steel-Dwass test after Kruskal-Wallis test, respectively; Fig. 5D-F). These results suggest that TRPM8 deficiency facilitates the development of EDs and more severe seizure events by elevating glutamate levels.

## Discussion

This study compared changes in extracellular glutamate concentrations following the administration of a seizure inducer and TRPM8 agonist in WT and TRPM8-KO mice. Our research yielded three key findings. First, as compared to the WT mice, mice with TRPM8 deficiency had a greater increase in glutamate levels triggered by the seizure inducer. The elevated glutamate levels were associated with more severe ED power and higher seizure scores. Second, the TRPM8 agonist WS-3 regulated glutamate levels, leading to anti-seizure-like effects, but these effects were absent in the TRPM8-KO mice. Third, with or without the observed anti-seizure-like effects, the glutamate levels after seizure inducer injection were positively correlated with beta-band power in the electrocorticograms (ECoGs) and with seizure severity following TRPM8 agonist injection. Our results suggest that TRPM8 agonists suppress the development of EDs and ESs by modulating extracellular glutamate levels, supporting previous studies indicating that TRPM8-expressing neurons in LS and POA are inhibitory GABAergic cells [13] and that TRPM8 agonists suppress excitatory post-synaptic glutamatergic neurons [14].

We observed that decreases in extracellular glutamate levels due to TRPM8 agonist administration were positively correlated with reduced ECoG power and seizure severity. This supports the long-standing hypothesis that an imbalance between excitation and inhibition underlies both ictogenesis and epileptogenesis. Excessive increases in extracellular glutamate levels can provoke epileptic seizures [31–35]. Since TRPM8-expressing neurons in LS and POA are inhibitory GABAergic [13], our findings suggest that TRPM8 agonists may suppress seizure events by regulating glutamate levels. Human brain microdialysis data align with prior animal studies in which glutamate levels were increased after seizure inducer injections into the hippocampus or administered intraperitoneally [36–40]. Conversely, other studies have reported a lack of correlation between excitatory neurotransmitter concentrations and seizure severity [12]. These conflicting results may arise from differences in terms of the locations of seizure foci and the recording methods. One study noted that microdialysis probes inserted into very small areas could not accurately represent glutamate concentrations across the entire focal region [12]. In contrast, our recordings were localized to the seizure inducer focus. Differences in temporal resolution may also explain the discrepancies. Microdialysis typically has a temporal resolution of several tens of minutes, and prior studies have shown that a lower temporal resolution can fail to detect transient glutamate level changes [41]. In our study, the glutamate levels in the cerebral cortex were recorded in real-time using a biosensor with a temporal resolution of seconds [15]. Taken together, these findings suggest that real-time, high-resolution glutamate recordings at representative seizure foci may help predict seizure severity in epilepsy.

Our results demonstrate that seizure-inducer-mediated increases in extracellular glutamate levels caused EDs and ESs. The TRPM8 agonist WS-3 decreased or reduced these increases in extracellular glutamate levels induced by the seizure inducer. In TRPM8-KO mice, neither reduction of glutamate levels was observed, even when the TRPM8 agonist was injected before or after the seizure inducer. In rodents, TRPM8 channels are expressed in the LS and POA, and to a lesser extent, they are expressed in the cerebral cortex [8]. LS and POA neurons are inhibitory neurons that express high levels of TRPM8 channels [13] that regulate cortical activity. Our data and these findings suggest that TRPM8 agonists decrease extracellular glutamate levels. Our results are consistent with electrophysiological experiments from previous studies, showing that the TRPM8 and TRP Ankyrin 1 (TRPA1) agonist Icilin reduced the amplitude of primary afferent stimulation-evoked potentials in lamina I and II neurons [14]. However, our data conflict with those in a previous study reporting that Icilin increased glutamate levels in the dorsal striatum [42]. This discrepancy may be attributed to the influence of TRPA1 channels, as they also affect extracellular glutamate levels. Thymol, another TRPM8 and TRPA1 agonist, has been shown to increase glutamate release in lamina II neurons by activating TRPA1 channels, suggesting that Icilin may increase extracellular glutamate levels through similar mechanisms [43]. Additionally, transient receptor potential (TRP) channel agents have multiple mechanisms of action, which could complicate the effects of TRPM8 activation on ESs and glutamate levels. Menthol and Icilin, for example, modulate Na^+^ channels, GABA_A receptors, and TRP-independent pathways [44–47]. These previous reports are consistent with our finding that the administration of WS-3 in TRPM8-KO mice decreased glutamate levels (Fig. 3B). In TRPM8-KO mice, the reductions of WS-3 on glutamate levels were observed at 5 and 20 min after the period of glutamate level reduction in WT mice, and WS-3 did not decrease the ED power during these two periods. These results suggested that the anti-seizure effects of WS-3 through TRPM8 channel-independent pathways were minor. In addition, anesthesia may influence extracellular glutamate levels, as urethane and sevoflurane affect the severity of EDs and ES [48]. Future electrophysiological studies are needed to clarify the direct mechanisms by which TRPM8 agonists exert their anti-seizure effects.

Our data showed that TRPM8 deficiency caused a further increase in extracellular glutamate levels, exacerbating EDs and ESs when compared to WT mice. These results align with previous reports indicating that TRPM8 deficiency leads to severe EDs, ESs, and febrile seizures [5, 49]. Given that TRPM8-expressing neurons in the rodent brain are inhibitory GABAergic cells [13], TRPM8 deficiency could be an exacerbating factor in seizure owing to the elevations in glutamate levels. However, whether these exacerbations extend to individual cell units remains to be investigated, warranting further studies involving TRPM8-KO mice and electrophysiological techniques.

Taken together, our study suggests that TRPM8 deficiency exacerbates seizure events, while TRPM8 agonists alleviate the severity of EDs and ESs by modulating extracellular glutamate levels. This indicates that TRPM8 channels may represent a viable therapeutic target for epilepsy. Because an acute seizure model and seizure inducer injections were used in this study, whether the anti-seizure mechanisms of action of TRPM8 agonists will translate to chronic seizure models remains unclear. Furthermore, whether the anti-seizure mechanisms of TRPM8 agonists will translate to patients with intractable epilepsy is unclear. Additionally, because anesthesia affects EDs and ESs, evaluating the efficacy of TRPM8 agonists on seizure model mice under awake condition will further clarify whether TRPM8 agonist suppresses focal EDs and seizures by regulating extracellular glutamate concentrations. As our findings suggest that TRPM8 agonists suppress the development of EDs and ESs by regulating glutamate levels, further research into the underlying mechanisms of TRPM8 agonists and TRPM8 deficiency could deepen our understanding of epilepsy’s pathophysiology.

## Supplementary information

Figure S1

Figure S2

## Data Availability

The raw data supporting the conclusion of this article will be made available by the corresponding author, without undue reservation. The original contributions presented in the study are included in the article, further inquiries can be directed to the corresponding author.
