# Supplementary material for: Anti-seizure effects of WS-3, a TRPM8 agonist, on focal onset seizure mouse model via reduction of extracellular glutamate levels
Source: Neuropsychopharmacology. 2025 Jun 26;50(12):1855–63. doi: 10.1038/s41386-025-02143-x (PMC12518671; doi:10.1038/s41386-025-02143-x)
Supplement: Supplementary file 1 — Figure S1 [file 41386_2025_2143_MOESM1_ESM.pdf]

Supplementary information

Fig. S1

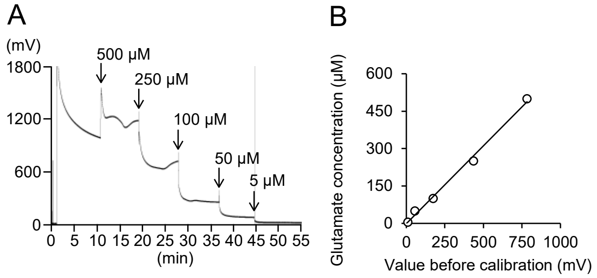

Figure S1. Calibration of extracellular glutamate levels from voltage levels to concentration using standard solutions.
(*A*) Voltage levels of extracellular glutamate (standard solutions: 5, 50, 100, 250, and 500 μM) were recorded using a biosensor. (*B*) Correlation diagram presenting the results of the preliminary verification. Standard solutions allowed for the accurate conversion of voltage levels into glutamate concentrations. The linear functions were $y = 0.624x - 0.997$ ($R^2 = 0.9951$, Pearson's correlation coefficients).
