# Supplementary material for: Anti-seizure effects of WS-3, a TRPM8 agonist, on focal onset seizure mouse model via reduction of extracellular glutamate levels
Source: Neuropsychopharmacology. 2025 Jun 26;50(12):1855–63. doi: 10.1038/s41386-025-02143-x (PMC12518671; doi:10.1038/s41386-025-02143-x)
Supplement: Supplementary file 2 — Figure S2 [file 41386_2025_2143_MOESM2_ESM.pdf]

Supplementary information

Fig. S2

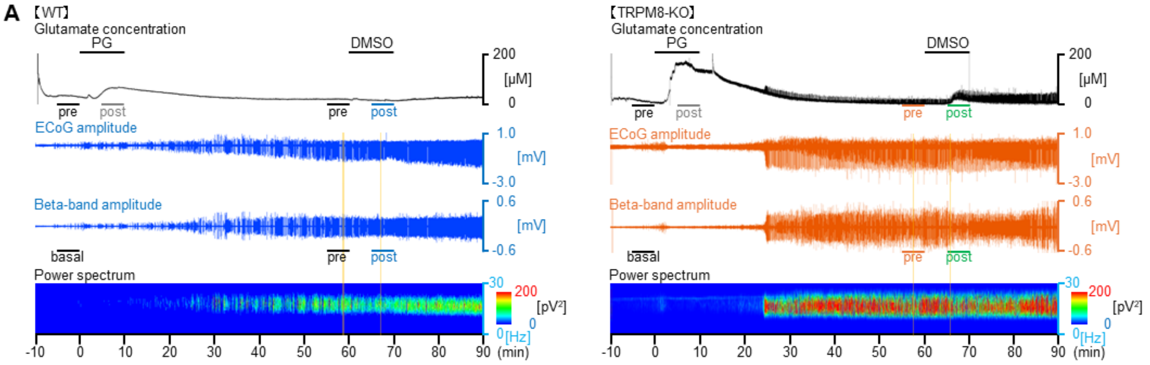

Figure S2. Effects of the DMSO on extracellular glutamate levels and beta-band power, after epilepsy inducer, Penicillin G potassium, injection.

(*A*) Representative changes in glutamate levels, ECoG amplitudes, Beta-band amplitudes, and beta-band power in wild-type (WT) and TRPM8 knockout (TRPM8-KO) mice. ECoG, electrocorticogram; DMSO, dimethyl sulfoxide; PG, Penicillin G potassium; TRPM8, transient receptor potential melastatin 8; TRPM8-KO, *TRPM8* homozygous knockout; WT, wild-type.
